# Awareness of Age-Related Changes Among Middle-Aged and Older Adults: Longitudinal Trajectories, and the Role of Age Stereotypes and Personality Traits

**DOI:** 10.3389/fpsyt.2022.902909

**Published:** 2022-05-25

**Authors:** Markus Wettstein, Anna E. Kornadt, Hans-Werner Wahl

**Affiliations:** ^1^Department of Psychology, Humboldt-Universität zu Berlin, Berlin, Germany; ^2^Department of Psychology, Heidelberg University, Heidelberg, Germany; ^3^Department of Cognitive and Behavioral Sciences, University of Luxembourg, Luxembourg, Luxembourg

**Keywords:** age stereotypes, personality, neuroticism, midlife, old age, views on aging

## Abstract

Awareness of Age-Related Change (AARC) describes to what extent people become aware of changes which they attribute to getting older. So far little is known regarding how different AARC dimensions change over time, to what extent these changes in different domains of AARC gains and losses are interrelated, and which predictors account for inter-individual differences in within-person longitudinal trajectories. Specifically, the extent to which individuals perceive age-related gains and losses might be shaped by their chronological age, their personality as well as by their general views on aging (i.e., their age stereotypes). We investigated changes in global and domain-specific AARC gains and losses over about five years in a sample of originally *N* = 423 participants aged 40 to 98 years at baseline. We analyzed the role of personality traits and age stereotypes for levels and changes of AARC, taking into account participants' age at baseline and controlling for gender, education, and subjective health. Based on longitudinal multilevel regression models, we observed mean-level declines in most AARC gain domains. In contrast, perceived general AARC losses, as well as AARC losses in health and physical functioning, in cognitive functioning and in social-cognitive/socio-emotional functioning remained, on average, stable over time. Baseline scores on AARC gains (global scale) were higher among individuals with higher neuroticism, openness, conscientiousness and more positive age stereotypes. Additionally, the association of higher neuroticism with higher AARC gain scores was stronger among individuals with more positive age stereotypes. Higher neuroticism and more negative age stereotypes also predicted higher baseline scores on AARC losses (global scale). At the same time, higher neuroticism was associated with a steeper decrease in AARC loss perceptions over time. Most of the intercorrelations within the intercepts and within the intra-individual trajectories of the different AARC domains were positive, but small in size. Our findings show the importance of considering trajectories of age-related gains and losses in parallel and across multiple developmental domains when investigating the subjective perception of the aging process. They also suggest that personality traits and general age stereotypes are related with individual experiences of aging.

## Introduction

Aging is a process that is continuously ongoing throughout the life span. However, how people subjectively perceive their aging process is by no means deterministically related to the chronological passing of lifetime. The concept “Views on aging” describes how individuals perceive, evaluate and interpret aging (1–4), encompassing both how they view aging or older adults in general (general views on aging; e.g., age stereotypes) but also their own aging (personal views on aging; e.g., self-perceptions of aging). Views on aging are inherently multidimensional in nature (5), comprising various domains, facets and constructs. Examples are subjective age, attitude toward own aging (6) or aging-related cognitions (7). These measures are interrelated, but distinguishable, and they reveal distinct associations with general dispositions (such as optimism, well-being, or health; (8). Generally, more positive views on aging are associated with various beneficial developmental outcomes, including greater well-being, better health and greater longevity (9–13). Therefore, it is important to study views on aging, their change and changeability over time, and their determinants, in order to derive interventions to promote favorable views on aging.

With the intention to enrich the conceptualization and measurement of views on aging, Diehl and Wahl (14) have, rather recently, proposed the concept “Awareness of age-related change” (AARC), which “refers to a person's state of awareness that his or her behavior, level of performance, or way of experiencing life has changed as a consequence of having grown older” [(14), p. 342]. Other than many of the existing concepts and operationalizations of views on aging which are unidimensional—and thus fall short in reflecting the inherent multidimensionality of lifespan development (15)- AARC explicitly takes into account the multidimensionality of views on aging. Specifically, AARC includes perceived age-related changes in five major developmental domains, namely health and physical functioning, cognitive functioning, interpersonal relations, social-cognitive and social-emotional functioning, as well as lifestyle and engagement. The conceptualization of these five domains was empirically supported in a qualitative-quantitative analysis based on a diary study (16). Moreover, different domains of AARC gains and losses differ in their relations with other variables such as age, well-being, or other views on aging constructs (17).

Informed by the principle of life span psychology that human development at any point in ontogenesis comprises both gains and losses (15, 18), AARC also differentiates between perceived gains vs. losses in each of the five domains. The AARC framework thus allows for a differential analyses of change in multiple AARC domains: In line with the general multidirectionality of lifespan development (15), individuals might perceive increasing age-related losses in certain developmental domains (such as health or cognitive functioning) when getting older, whereas other domains (such as interpersonal relations or social-cognitive and social-emotional functioning) might not necessarily reveal a trend of increasing losses, and even generate gains, with increasing age.

Of note, AARC is not the only views on aging concept that takes a multidimensional perspective, but its differentiation of five domains of gains and losses is unique. Another multidimensional construct of views on aging are the “personal experiences of aging” conceptualized by Steverink et al. (7), which are frequently referred to as “AgeCog scales” [aging-related cognitions (3, 4)] and comprise the dimensions physical decline, social loss, and continuous growth. Similarly, Laidlaw et al.'s (19) Attitudes Toward Aging Questionnaire consists of three subscales, namely psychological growth, psychosocial loss, and physical change. Finally, tapping into eight different life domains in which age stereotypes and future self-views are rated, Kornadt and Rothermund (20) also proposed a multidimensional measure of views on aging. However, longitudinal studies on *changes* in these measures are still rare.

A considerable number of studies shows that individuals' awareness of their aging is related to outcomes of health and functioning. For instance, higher scores on AARC gains and lower scores on AARC losses are associated with better physical and mental health (17, 21–28). Moreover, higher AARC losses—but also higher AARC gains—are cross-sectionally associated with poorer cognitive performance (29, 30). The authors discuss this unexpected finding by speculating that individuals with poorer cognitive functioning might be “paying more attention to their cognitive gains as this may facilitate acceptance of negative changes and re-establishment of self-efficacy and positive emotional states” (30). However, as these findings are cross-sectional, no firm causal conclusions can be drawn. Higher scores on AARC losses are also associated with greater pain (31). Moreover, greater perceived age-related gains as well as fewer perceived age-related losses are meaningfully related to a lower mortality risk in late life, and these effects remained robust when controlling for health, socio-demographic indicators as well as psychosocial factors (32). The AARC construct thus seems to have a unique and robust prognostic effect on outcomes such as mortality.

Given these findings and the role of change as an inherent component of AARC, a better understanding of how AARC develops across the second half of life, and which factors predict AARC developmental trajectories is of high importance. So far, however, little is known about age-related changes in AARC, particularly regarding differential age-related trajectories across the specific AARC subdomains. Moreover, determinants of AARC and of AARC changes have rarely been empirically addressed and could hardly be identified so far. To give one example, whereas AARC has been found to longitudinally predict depressive symptoms, the opposite effect, from depressive symptoms to AARC, could not be empirically supported (23, 24). In this study, we will thus investigate AARC changes over approximately 5 years in middle-aged and older adults by taking a multidimensional perspective and contrasting trajectories of gains and losses in all 5 AARC domains. We will also investigate the extent to which the AARC dimensions and their age-related changes are interrelated and whether “de-differentiated,” strongly interrelated changes across AARC domains—e.g., a general trend of decreasing gains and increasing losses across all domains—can be observed. Finally, we will analyze the role of age stereotypes and personality, which—according to the conceptual AARC framework proposed by Diehl et al. (33)—represent proximal and distal AARC antecedents, as well as of their interaction for age-related changes in general AARC gains and losses.

### Developmental Trajectories of AARC

Most studies investigating the relationship of AARC with chronological age have thus far been cross-sectional. Some of the available cross-sectional evidence suggests that both AARC gains and losses reveal a positive association with chronological age among samples of middle-aged and older adults, with stronger age associations for losses than for gains (17, 22). With increasing age, individuals thus seem to perceive more age-related changes both on the gain side and on the loss side, which could indicate a general age-related increase in sensitivity for perceiving age-related change. However, Sabatini et al. (34) found in their study that when controlling for socio-demographic variables, only AARC losses are positively related with age, whereas the association of AARC gains with age is negative.

Generally, such cross-sectional associations of AARC with age might to some extent reflect cohort differences rather than age effects. Moreover, findings from cross-sectional studies cannot reveal whether *change* in AARC varies according to age. For instance, as cognitive and health-related resources decrease from midlife to old age, a steeper increase of perceived losses in these domains in in late life than in midlife can be assumed (35). Therefore, it is unfortunate that the nature and extent of age-related *change* in AARC across midlife and old age from a genuinely longitudinal perspective has so far found limited empirical attention.

One of the very few available longitudinal studies found that in a German sample of more than 900 adults aged 80 years and older, AARC gains decreased, whereas AARC losses increased within a 2 year time period (35). However, these change trends may be specific to the life phase of very old age, which is generally characterized by a heightened (physical) vulnerability and closeness to death (36, 37). Diehl et al. (33) investigated changes in aging-related cognitions—which reveal some conceptual overlap with AARC—in the second half of life. They found a mean-level increase in physical decline and in social loss, as well as a mean-level decrease in continuous growth. All these mean-level changes were non-linear, with steeper changes toward losses in old and very old age.

In a previous contribution (38), we analyzed trajectories of AARC based on the same data set that will be used in the following analyses, but without a distinction of the AARC subdomains. Given that 2020 was the time of the outbreak and spreading of COVID-19 in Germany, Wahl et al. (38) empirically differentiated between age-related and pandemic-related change in AARC. AARC-gains and losses both significantly decreased between 2012 and 2017, potentially reflecting age-related change, whereas they increased between 2017 and 2020, which might have been, at least to some extent, due to the outbreak of the pandemic in 2020.

In conclusion, knowledge regarding age-related changes in AARC is very limited, with findings restricted to the general domains of AARC losses and gains without subdomain-specific differentiations. However, following the principles of multidimensionality and multidirectionaliy of life span development in general (15), it is very plausible that gains and losses of the different AARC subdomains also differ with regard to (direction and rate of) change. For instance, health and (fluid) cognitive abilities typically decline with advancing age (39–44), which might result in an age-related increase in perceived AARC losses in these domains. At the same time, more differentiation might be needed, as crystallized cognitive abilities, unlike fluid ones, remain stable or even increase from midlife on and do not decline before very old age [e.g., (45, 46)]. Similarly, objective health declines in old and very old age, whereas subjective perceptions of health seem to remain stable even in the oldest-old (47). It is thus highly plausible that perceived age-related gains and losses in domains such as cognitive functioning or health co-occur.

### AARC Trajectories From a Multidimensional and Multidirectional Perspective

With regard to the emergence of AARC and the potential onset of AARC changes in the second half of life, Diehl et al. (48) state that “middle-aged adults (…) are at a life stage when they become aware of age-related changes” (p. 583). Generally, middle adulthood seems to be “point in life when vulnerabilities begin to emerge” (49). Similarly, according to Westerhof and Wurm (3), midlife “marks the shift from the predominance of growth and gains to an increasing risk of age-related losses” (p. 152). Therefore, increases in perceived age-related losses can be expected from midlife on. However, as not all perceived age-related changes are necessarily negative, and since late midlife might be a time in which changes—positive as well as negative ones—are particularly frequently attributed to aging (50), it is possible that not only perceived loss- but also age-related gains increase in midlife. Examples for potential age-related gains are interpersonal relations as well as socio-cognitive and social-emotional functioning, which do not necessarily reveal negative trends with advancing age; rather, emotionally meaningful relationships get more important with advancing age (51), and the family network remains stable from adolescence into old age, whereas the friendship network as well as other networks decrease (52). Also, according to empirical evidence, social relationships get more positive across adulthood (53), and the extent to which social needs are fulfilled is very similar between different age groups ranging from emerging adults to oldest-old individuals (54). Individuals may invest more resources in interpersonal relations when getting older and, in consequence, may even perceive gains in this domain. At the same time, developmental gains and losses can co-occur (15), and AARC gains and losses have been found to be positively interrelated (21, 22). Older adults might thus not only perceive more gains, but also—due to factors such as widowhood, death of age peers etc- more losses in interpersonal relations compared to middle-aged adults. With regard to socio-cognitive and socio-emotional functioning, certain aspects or skills such as affect regulation and emotional stability seem to improve with age (55, 56), so that individuals might in consequence perceive more gains in this domain when getting older.

These examples underline the importance of distinguishing between different AARC domains when investigating age-related change in perceived AARC gains and losses. In addition, given that depending on the specific domain, changes might be more or less likely attributed to age (50), a domain-specific perspective on AARC change is imperative. To additionally analyze to what extent change dynamics in AARC are indeed multidirectional, or rather follow a trend of dedifferentiation and general decline– as has been observed in cognitive aging research with regard to changes in different cognitive abilities and their couplings (57, 58)—we will also investigate the associations between levels and changes of the different AARC subdomains.

### Personality and Age Stereotypes as Antecedents of AARC

According to the theoretical framework developed by (59), both distal and proximal antecedents shape an individual's awareness of age-related change. Previous findings (22, 50, 60) show the particular relevance of two of these antecedents, namely personality (distal antecedent) and age stereotypes (proximal antecedent) for AARC. In addition, both personality as well as age stereotypes might affect the general likelihood of experiencing life events and changes—which might be interpreted as age-related— (61, 62).

#### Personality Traits as Distal Antecedents of AARC

Personality traits, such as the Big Five (63, 64) affect individuals' attitudes and mindset and might therefore also have an impact on how individuals perceive their aging. This is most obvious for neuroticism as a general disposition of emotional instability and a tendency to worry. Higher neuroticism might be associated with a greater focus on perceived age-related losses than on age-related gains. It might also promote the experience of losses since individuals scoring high on neuroticism tend to have suboptimal, passive and less effective coping strategies (65, 66), potentially also when coping with age-related changes and challenges. Greater neuroticism is also positively associated with physical symptom reporting (67), which might in turn trigger perceptions of age-related losses.

Regarding the other Big Five traits, a higher openness to experience could also include a higher openness toward the aging process *per se* and its ambiguity, so that not every perceived age-related change might be perceived as negative among individuals with high openness scores. Greater extraversion and agreeableness are associated with characteristics of social networks [such as larger friend network size; higher felt closeness to confidants; (68)] and also with adaptive coping strategies such as support seeking, positive reappraisal or problem-focused coping (65, 66), which might be also helpful when facing age-related changes. Finally, higher conscientiousness, a trait associated with better health behaviors, better health and longevity (69–71), might help individuals to influence their aging in a way that results in perceptions of fewer age-related losses and of more age-related gains. In conclusion, extraversion, openness, agreeableness and conscientiousness are expected to promote the perceptions of age-related gains and to be inversely associated with perceived age-related losses.

With regard to empirical evidence, Kornadt et al. (72) investigated longitudinal associations between personality traits and attitudes toward own aging in middle-aged and older adults across a time period of 20 years. They found that lower neuroticism, higher conscientiousness, as well as higher openness were predictors of more positive attitudes, whereas the effect of extraversion varied in direction over time. Shenkin et al. (73) investigated personality predictors of attitudes to aging (psychosocial loss, physical change, psychological growth) in older adults. Greater neuroticism predicted greater psychosocial loss. All three attitudes to aging dimensions were more positive among individuals scoring higher on extraversion, agreeableness and conscientiousness. Higher openness scores were related with more positive perceptions of physical change and psychological growth. Apart from physical change perceptions, which were most closely related with indicators of physical function, personality traits contributed—together with anxiety and depression—to the greatest proportion of variance in attitudes to aging accounted for, with their predictive impact exceeding the one of socio-demographic variables as well as of cognitive and physical functioning.

With regard to AARC, Rupprecht et al. (60) investigated the role of personality traits for AARC trajectories across 4.5 years in adults aged 40 to 98 years. Their analyses yielded a positive cross-sectional association between neuroticism and AARC losses, but neuroticism was, besides openness and conscientiousness, also positively associated with AARC gains. Longitudinally, only higher conscientiousness was a predictor of decreases in AARC losses over time. In the current study, we use the same data set as analyzed by Rupprecht et al. (60), but extend their findings by investigating more measurement occasions, by taking a domain-specific, multidimensional and –directional perspective on AARC changes and also by considering the role of age stereotypes for AARC, in addition to personality. Also, potential interactions of personality with age stereotypes as predictors of AARC trajectories will be investigated.

#### Age Stereotypes as Proximal Antecedents of AARC

Beliefs and perceptions about older people and the aging process in general are of considerable significance in determining whether change is perceived as age-related or not (50). Age stereotypes serve as expectations against which changes are interpreted. Thus, if persons believe that a certain characteristic will change as a function of age, they are more likely to attribute experienced changes to age. Besides, according to stereotype embodiment theory (74) and according to the conception of “age stereotype internalization/contamination” (75, 76) as well as other conceptual frameworks [e.g., (4)], age stereotypes are internalized and become increasingly self-relevant with advancing age. When growing older, individuals become targets of their own age stereotypes so that their age stereotypes might turn into a self-fulfilling prophecy. Therefore, it is very likely that negative age stereotypes predict fewer perceptions of age-related gains as well as less favorable change of these perceptions, whereas they are associated with more perceived age-related losses and an increase thereof over time. Indeed, based on longitudinal data with a sample of German and US middle-aged and older adults, Brothers et al. (22) found that negative age stereotypes predicted greater perceived age-related losses as well as fewer perceived age-related gains ~2.5 years later. AARC gains and losses also seemed to mediate associations of age stereotypes with subsequent mental and physical health.

Regarding potential interactions between personality and age stereotypes, we refer again to the theoretical framework by Diehl et al. (59), which postulates that distal and proximal antecedents of AARC may interact. Therefore, we will also investigate interactions between age stereotypes and personality traits. For instance, it is possible that certain stereotype-personality constellations, such as negative age stereotypes combined with high neuroticism, are particularly predictive of greater perceived age-related losses and fewer age-related gains, whereas personality traits such as conscientiousness or openness might to some extent operate in a compensatory way, potentially counteracting the presumed detrimental impact of negative age stereotypes on awareness of age-related change. Furthermore, we will control for the additional categories of distal antecedents postulated in the (59) model, namely sociodemographic antecedents (age, gender, education) and biological/health-related antecedents (subjective health).

### The Present Study

Awareness of age-related change (AARC) is a rather recent construct that was found to be important for health and well-being in late life. Evidence with regard to AARC change and its determinants is still scarce. We will therefore investigate trajectories of the global AARC gain and loss scales as well as of different AARC gain and loss subdimensions over approximately 5 years in a sample of middle-aged and older adults. Our study aims are

1. To describe trajectories of AARC-Gains and AARC-Losses in general as well as across the different subdomains (health and physical functioning, cognitive functioning, interpersonal relations, social-cognitive and social-emotional functioning, as well as lifestyle and engagement). Further, to determine the extent to which changes in AARC domains are interrelated and potentially dedifferentiated vs. multidirectional and independent of each other.
2. Following the conceptual framework by Diehl et al. (59), we also aim at investigating the role of personality traits and age stereotypes as distal and proximal antecedents for general AARC gains and losses as well as their changes (controlling for age, gender, education and subjective health). For these analyses, we focus solely on the two overarching AARC scales rather than on the gain and loss subdomains. We decided for this approach in order to limit the number of statistical models, to avoid the risk of Type I error inflation due to multiple testing, to reduce the complexity of results, as well as for the sake of comparability with previous studies, which mostly used the broad AARC scales without a differentiation of subdomains. We expect that higher neuroticism scores might predict more perceived age-related losses *and* gains over time, whereas the other Big Five traits might be positively related with change in AARC gains, but negatively with change in AARC losses. Negative age stereotypes are expected to be related with perceptions of fewer age-related gains and of more age-related losses over time.

## Materials and Methods

### Sample

We used data from three measurement occasions (T1 in 2012, *n* = 423; T2 in 2015, *n* = 356; T3 in 2017, *n* = 299). The data were collected via paper-pencil questionnaires that were sent to the study participants with paid return service.

We decided not to include the fourth measurement occasion (T4 in June-September 2020, *n* = 233) for the following reasons: First, the COVID-19 outbreak in spring 2020 in Germany with its far-reaching consequences for individuals' lives, well-being and attitudes (77, 78) might have also affected how individuals perceive their own aging (38, 79). The “normative” age-related changes in AARC, which are of primary interest in this study, might thus be biased when including this peri-pandemic measurement occasion. Moreover, at T4, a short-form for the assessment of AARC was used [AARC-10 SF (25)], comprising only 10 items and thus not allowing for a differentiation of AARC subdomains. Finally, data were to a large extent (79%) collected online in 2020, whereas only paper-pencil questionnaires were administered from 2012 to 2017, which might have implications with regard to potential assessment mode effects that could bias estimates of AARC changes.

All individuals provided written informed consent prior to the study participation at each measurement occasion. Approval for Wave 4 of the longitudinal data used in this study was received from the Institutional Ethics Review Board of the Faculty of Behavioral and Empirical Cultural Sciences of Heidelberg University (AZ Wahl 2020 1/1). Wave 3 was approved by the same board with a letter dating from February 17, 2017 (no protocol no.). Waves 1 and 2 were approved by the Colorado State University (CSU) Institutional Review Board (IRB) protocol #10-2080H based on a formal cooperation between Heidelberg University and CSU. All study participants were informed that they could change their minds and withdraw their agreement at any time. Contact data of the participants were stored locally on a university computer as a data file with password protection. Participants were also informed that their data were used and analyzed solely for scientific purposes, and that their data were not shared with any third parties. Analyses of the data was done with personal identifiers removed. Data are archived for 10 years, in agreement with the recommendations of the German Research Foundation. This study was designed and organized in line with the rules and guidelines specified in the “Leitlinien zur Sicherung guter wissenschaftlicher Praxis,” 2019 [GOOD RESEARCH PRACTICE. Guidelines for Safeguarding Good Research Practice, 2019; access via: https://wissenschaftliche-integritaet.de/en/code-of-conduct/) by the German Research Foundation.

A sample description is provided in Supplementary Table 1. Study participants were 40–98 years old at baseline (M = 62.94 years, SD = 11.84 years). The majority of the sample (64.3%, *n* = 272) were women. Individuals participated up to three times (M = 2.55, SD = 0.71; individuals with one study participation: *n* = 54 [12.8%]; individuals with two study participations: *n* = 83 [19.6%]; individuals with three study rocher participations: *n* = 286 [67.6%]).

### Measures

#### Awareness of Age-Related Change (AARC)

A questionnaire comprising 50 items [AARC-50, comprising 25 gain-related items and 25 loss-related items (21)] was used for the assessment of AARC. All items begin with the stem “With my increasing age, I realize that...,” which is followed either by a gain-related (e.g., “…my friendships and relationships have become stronger”) or a loss-related statement (e.g., “…I am more forgetful”). Participants indicated their agreement to each statement on a 5-point response scale from 1 (=not at all) to 5 (=very much). Brothers et al. (21) found a 2-factor solution for this questionnaire, both based on exploratory and confirmatory factor analysis, with the gain and loss scales constituting one overarching gain and loss factor. They also demonstrated convincing psychometric properties of the instrument in terms of scale and item reliability, convergent and divergent validity, and predictive validity.

All gain-related and all loss-related items were combined into a mean score of general AARC gains (Cronbach's α T1–T3:0.92, 0.92, 0.93) and general AARC losses (Cronbach's α T1–T3:0.92, 0.92, 0.93), respectively. Moreover, as the questionnaire includes 10 items for each subdomain (gains/losses in health and physical functioning, cognitive functioning, interpersonal relations, social-cognitive/social-emotional functioning, and lifestyle and engagement), mean gains and losses score for each subdomain were computed as well (Cronbach's α gains and losses across all domains and measurement occasions are listed in Supplementary Table 1 and ranged from 0.67 to 0.87).

#### Personality

At the first measurement occasion, the Big Five personality traits were assessed based on the NEO Five Factor Inventory [NEO-FFI (80)]. Each trait was assessed by 12 items, with a 5-point response format (1 = completely untrue, 5 = completely true); Cronbach's α at T1: neuroticism 0.83; extraversion: 0.77; openness: 0.62; agreeableness: 0.69; conscientiousness:0.80.

#### Age Stereotypes

The multidimensional age stereotype scale (20) was used to measure age stereotypes. This assessment instrument differentiates between eight stereotype domains (physical and mental fitness, health, and appearance; family and partnership; friends and acquaintances; personality and way of living; work/employment; spirituality; finances; leisure). Each stereotype domain consists of 3–4 items. Every item comprises two opposing statements representing a stereotypic belief about older adults and is answered on an 8-point Likert scale in-between these statements. As an example, one of the items belonging to the friends and acquaintances subscale is “Older persons… (1) …have problems establishing new friendships” to “(8) …can easily establish new friendships.” As we were interested in the association of general, rather than domain-specific, age stereotypes with AARC and AARC changes, we computed a mean score of age stereotypes across all 25 items, resulting in a very high internal consistency (Cronbach's α at T1:0.90).

#### Covariates

We took sociodemographic and health-related antecedents of AARC, specified as distal antecedents in the (59) framework, into account by controlling for age, gender, education and subjective health in all analyses. Education was measured by years of schooling. Self-rated health was assessed by a single-item question (“How would you rate your health in general?”) with a 5-point response format ranging from 1 (*excellent*) to 5 (*poor*).

### Analyses

We computed longitudinal multilevel regression models (81, 82) to investigate the linear trajectories of general AARC gains and losses, as well as of domain-specific AARC gains and losses, controlling for age, gender, education, and subjective health. To test for non-linear associations of baseline age with the AARC domains at baseline as well as with AARC changes, we included an age^2^ term, which remained in the model only if it reached statistical significance. By using full information maximum likelihood (FIML) estimation, we made sure that all information was used for parameter estimations, including data from those study participants who dropped out of the study and who thus provided only one or two observations.

To quantify the extent to which the different AARC domains—and particularly their changes—are interrelated, we analyzed the correlations of the individual level and slope estimates, as obtained from the multilevel regression analyses, across all subdomains. Finally, we additionally included the Big Five personality traits, age stereotypes and their interactions as predictors of general AARC gains and losses as well as of their trajectories. Due to missing values on the covariates and on specific AARC items, the sample size for these analyses ranged from 397 (models including personality, age stereotypes, and all covariates) to 410 (models including all covariates).

## Results

### Trajectories of AARC Gains and Losses

**Figure 1:**
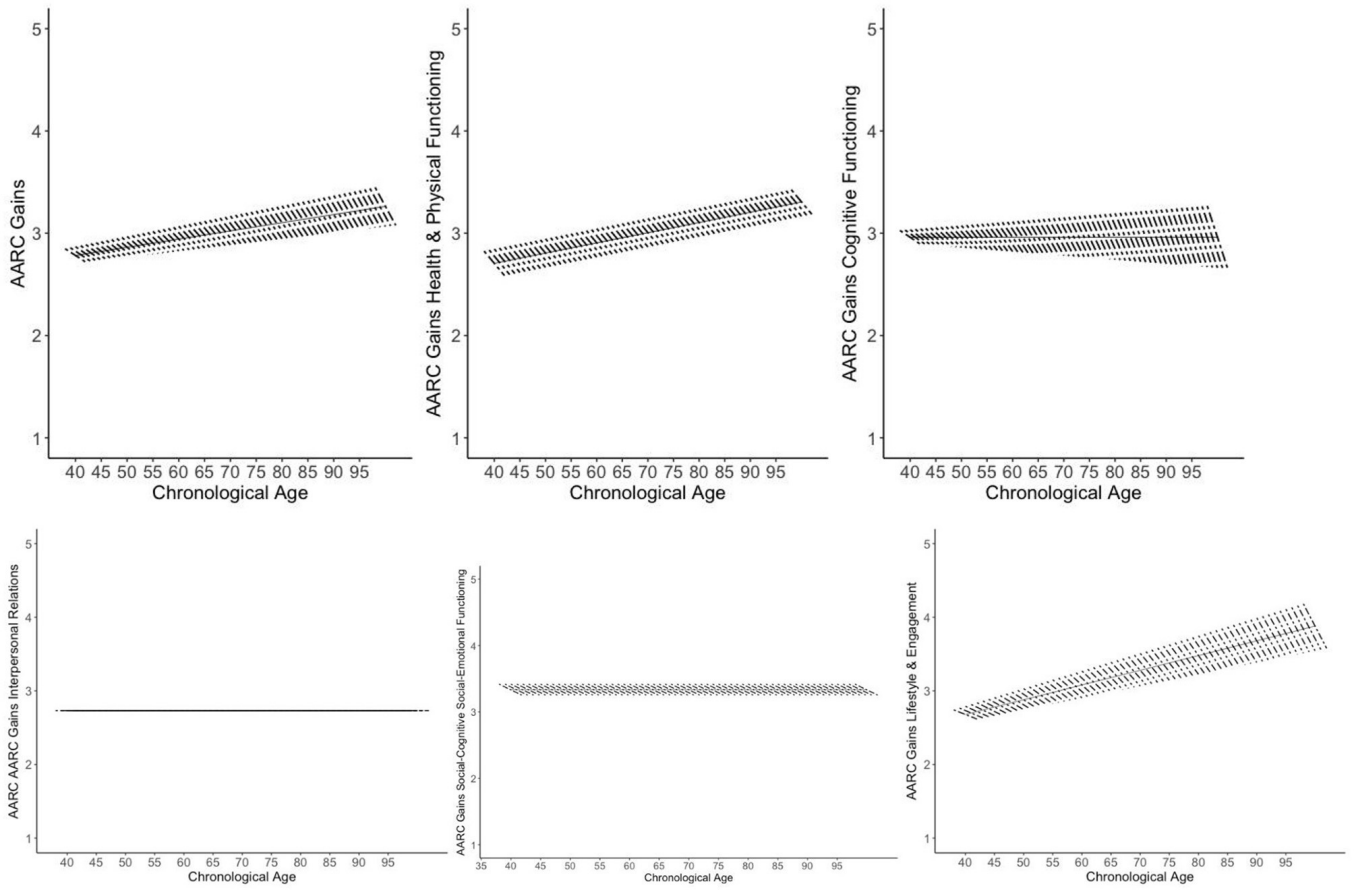
Trajectories of AARC gains. Figures adapted from Gerstorf et al. (84). The figure shows model-implied 5 year within-person changes in AARC gains by baseline age as short, thick lines. The single linear age trend in AARC gains is shown as long, thin line. Models are adjusted for gender, education, and self-rated health. The graphs illustrate a general trend of 5 year declines in AARC gains across the different domains (with the exception of interpersonal relations, which—on average—remained stable over 5 years). At the same time, baseline AARC gains in general as well as with regard to health and physical functioning were greater among older adults compared to middle-aged individuals. Gains in lifestyle and engagement at baseline were nonlinearly related with age (see Supplemental Figure 1).

**Figure 2:**
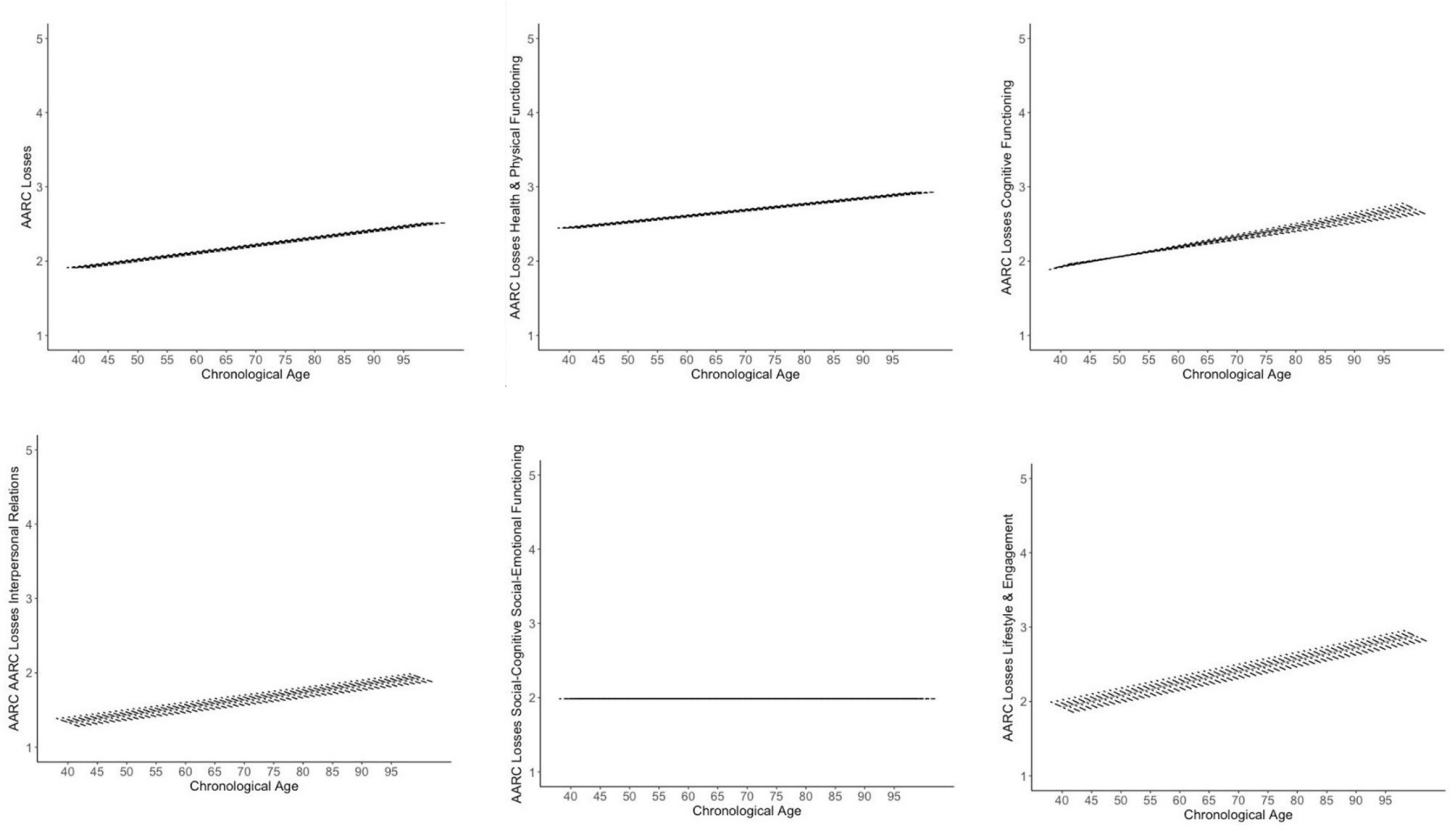
Trajectories of AARC losses. Figures adapted from Gerstorf et al. (84). The figure shows model-implied 5 year within-person changes in AARC losses by baseline age as short, thick lines. The single linear age trend in AARC losses is shown as long, thin line. Models are adjusted for gender, education, and self-rated health. The graphs illustrate a general trend of mean-level stability in AARC losses across the different domains, with the exception of losses in interpersonal relations and in lifestyle and engagement, which decreased over time. At the same time, perceived losses in general and across most domains at baseline were greater among individuals who were older. Only losses in socio-cognitive/social-emotional functioning were not related with age. The association of losses in interpersonal relations with age was non-linear (see Supplemental Figure 1).

Controlling for age, gender, education, and subjective health, the following mean-level changes in AARC gains and losses were identified (see Tables 1, 2 and Figures 1, 2): There was a mean-level decline in general AARC gains (by about 0.06 points per year), whereas mean-level change in general AARC losses was not significant (*p* =0.08), indicating overall stability across 5 years.

**Table 1:** Longitudinal multilevel regression models of changes in AARC gains.

**Model Estimates**	**AARC Gains (General)**	**AARC Gains Health and Functioning**	**AARC Gains Cognitive Functioning**	**AARC Gains Interpersonal Relations**	**AARC Gains Social-Cognitive/Social-Emotional Functioning**	**AARC Gains Lifestyle and Engagement**
**Fixed Regression Coefficients**
Intercept [*SE*]	2.961*** [0.053]	2.928*** [0.069]	2.960*** [0.060]	2.733*** [0.063]	3.062*** [0.069]	3.259*** [0.080
Age [SE]	0.008** [0.003]	0.010** [0.004]	0.005 [0.003]	0.005 [0.003]	−0.001 [0.004]	0.026*** [0.004]
Age^2^ [SE]						−0.001*** [0.000]
Gender [SE]	0.158* [0.066]	0.081 [0.087]	0.070 [0.075]	0.068 [0.079]	0.414*** [0.086]	0.166 [0.093]
Education [SE]	0.025 [0.017]	0.007 [0.022]	−0.001 [0.019]	0.009 [0.020]	0.064** [0.022]	0.044 [0.023]
Self-Rated Health [SE]	0.034 [0.039]	0.027 [0.051]	0.059 [0.045]	0.059 [0.047]	0.004 [0.051]	0.036 [0.055]
Linear slope 2012–2020 [*SE*]	−0.005*** [0.001]	−0.005*** [0.001]	−0.007** [0.001]	−0.001 [0.001]	−0.004** [0.001]	−0.007*** [0.002]
Age*Slope [SE]	−0.000* [0.000]	−0.000 [0.000]	−0.000* [0.000]	−0.000 [0.000]	−0.000 [0.000}	−0.000* [0.000]
Gender*Slope [SE]	0.001 [0.001]	−0.000 [0.002]	0.001 [0.002]	0.001 [0.002]	0.000 [0.002]	0.024 [0.002]
Education*Slope [SE]	−0.001* [0.000]	−0.000 [0.000]	−0.000 [0.000]	−0.000 [0.000]	−0.001** [0.000]	−0.001* [0.000]
Health*Slope [SE]	0.002** [0.001]	0.003** [0.001]	0.003** [0.001]	0.001 [0.001]	0.002* [0.001]	0.002 [0.001]
**Random Variances**
Variance Intercept [*SE*]	0.262*** [0.030]	0.373*** [0.053]	0.319*** [0.030]	0.368*** [0.042]	0.476*** [0.048]	0.465*** [0.059]
Variance Linear Slope [*SE*]	0.000 [0.000]	0.000 [0.000]	0.000 [0.000]	0.000 [0.000]	0.000** [0.000]	0.000 [0.000]
Cov. Intercept-Slope [*SE*]	0.001 [0.000]	0.000 [0.000]	n/a	−0.000 [0.001]	−0.001* [0.001]	0.000 [0.000]
Residual Variance [*SE*]	0.150*** [0.012]	0.340*** [0.027]	0.213*** [0.014]	0.220*** [0.018]	0.208*** [0.017]	0.345*** [0.028]
BIC	21,788.8	2,463.4	2,105.0	2,199.9	2,247.4	2,615.5
*R* ^2^	0.117	0.086	0.167	0.094	0.209	0.143

*Time unit is months (since 2012). R^2^ was computed according to Xu (83). AARC = Awareness of age-related change*.

*p <0.05; **p <0.01; ***p <0.001.

**Table 2:** Longitudinal multilevel regression models of changes in AARC losses.

**Model Estimates**	**AARC Losses (General)**	**AARC Losses Health and Functioning**	**AARC Losses Cognitive Functioning**	**AARC Losses Interpersonal Relations**	**AARC Losses Social-Cognitive/Social-Emotional Functioning**	**AARC Losses Lifestyle and Engagement**
**Fixed Regression Coefficients**
Intercept [*SE*]	2.135*** [0.042]	2.624*** [0.062]	2.218*** [0.063]	1.500*** [0.045]	1.986*** [0.054]	2.280*** [0.055]
Age [SE]	0.010** [0.002]	0.008* [0.003]	0.013*** [0.003]	0.007** [0.002]	0.004 [0.003]	0.016*** [0.002]
Age^2^ [SE]				0.000*** [0.000]		
Gender [SE]	0.082 [0.053]	0.030 [0.077]	0.0.090 [0.079]	−0.051 [0.052]	0.190** [0.067]	0.154* [0.069]
Education [SE]	−0.003 [0.013]	0.031 [0.019]	−0.002 [0.020]	−0.023 [0.013]	−0.016 [0.017]	−0.005 [0.017]
Self-Rated Health [SE]	0.347*** [0.031]	0.592*** [0.046]	0.239*** [0.047]	0.248*** [0.031]	0.277*** [0.040]	0.367*** [0.041]
Linear slope 2012–2020 [*SE*]	−0.001 [0.001]	0.000 [0.001]	−0.001 [0.001]	−0.002* [0.001]	−0.001 [0.001]	−0.003** [0.001]
Age × Slope [SE]	−0.000 [0.000]	−0.000 [0.000]	−0.000* [0.000]	−0.000 [0.000]	−0.000 [0.000]	−0.000 [0.000]
Gender × Slope [SE]	−0.000 [0.001]	0.000 [0.002]	−0.002 [0.001]	0.002 [0.001]	−0.001 [0.001]	−0.000 [0.001]
Education × Slope [SE]	−0.000 [0.000]	−0.001 [0.000]	0.000 [0.000]	0.000 [0.000]	−0.000 [0.000]	−0.000 [0.000]
Health × Slope [SE]	−0.001 [0.001]	−0.003* [0.001]	– 0.000 [0.000]	−0.000 [0.001]	−0.000 [0.001]	0.000 [0.001]
**Random Variances**
Variance Intercept [*SE*]	0.166*** [0.019]	0.298*** [0.042]	0.432*** [0.041]	0.153*** [0.019]	0.243*** [0.032]	0.272 *** [0.032]
Variance Linear Slope [*SE*]	0.000 [0.000]	0.000 [0.000]	0.000* [0.000]	0.000* [0.000]	0.000 [0.000]	0.000 [0.000]
Cov. Intercept-Slope [*SE*]	0.000 [0.000]	0.000 [0.001]	−0.001* [0.001]	−0.000 [0.000]	−0.000 [0.001]	−0.000 [0.001]
Residual Variance [*SE*]	0.094*** [0.008]	0.265*** [0.021]	0.150*** [0.012]	0.103*** [0.008]	0.188*** [0.015]	0.178*** [0.014]
BIC	1,333.0	2,291.9	1.955.5	1,381,0.4	1,959.5	1,906.6
*R* ^2^	0.105	0.104	0.144	0.127	0.099	0.093

*Time unit is months (since 2012). R^2^ was computed according to Xu (83). AARC = Awareness of age-related change.*

*p <0.05; **p <0.01; ***p <0.001.

With regard to gain subdomains, AARC gains in health and physical functioning declined, on average, over time (by about 0.06 points per year). Also, AARC gains in cognitive functioning revealed a significant mean-level decline (by about 0.08 points per year), whereas AARC gains in interpersonal relations remained, on average, stable over 5 years. Mean-level declines in AARC gains regarding social-cognitive/social-emotional functioning (by about 0.04 points per year) and regarding lifestyle and engagement (by about 0.08 points per year) were both significant.

Regarding subdomains of AARC losses, there was no significant change in perceived age-related losses of health and physical functioning as well as in social-cognitive/social-emotional functioning. Also, perceptions of losses in cognitive functioning remained, on average, stable over time. Perceived losses in interpersonal relations revealed a significant mean-level decline, which amounted to an annual decrease of about 0.02 points. Also, perceived losses in lifestyle and engagement significantly declined by, on average, 0.04 points per year.

In terms of covariates, age was positively associated with general baseline AARC gains and with gains in health and functioning. Age was non-linearly related with gains in lifestyle and engagement at baseline, which were highest among individuals in early-old age, but lower in middle-aged and very-old adults (see Supplementary Figure 1). Apart from these cross-sectional associations of age at baseline with AARC scores at baseline, an older age was also significantly related with a steeper longitudinal decline in general AARC gains as well as in gains in cognitive functioning and in lifestyle and engagement over time. Individuals who were older also had higher baseline general AARC loss scores and higher baseline loss scores in health and functioning, cognitive functioning, as well as lifestyle and engagement. The association of age with baseline AARC losses in interpersonal relations was non-linear (see Supplementary Figure 1), with particularly high baseline loss scores among the oldest-old.

Women had higher scores on general AARC gains at baseline as well as on gains in social-cognitive/social-emotional functioning than men. However, they also scored higher on baseline AARC losses in in social-cognitive/social-emotional functioning, and in lifestyle and engagement than men. Rate of change in AARC gains and losses, both for the general scales as well as across the different AARC subdomains, did not differ by gender.

More years of education were associated with a steeper decline in general AARC gains and in gains regarding social-cognitive/social-emotional functioning as well as lifestyle and engagement. At the same time, higher education was related with higher baseline gain scores in social-cognitive/social-emotional functioning.

Finally, a poorer subjective health was associated with less decline in general AARC gains and in gains related to health, cognitive functioning, and social-cognitive/social-emotional functioning. However, the association of subjective health with AARC seems to be complex, as all baseline AARC loss scores—with the exception of those referring to interpersonal relations—were higher among those reporting poorer health, although poorer health was also associated with steeper longitudinal decreases in AARC health losses.

Overall proportional reduction in residual variance (computed according to Xu (83)) across the models ranged from 0.09 ≤ R^2^ ≤ 0.21 for all AARC gain domains and from 0.09 ≤ R^2^ ≤ 0.14 for all AARC loss domains.

### Intercorrelations of AARC Levels and Changes

Regarding the intercorrelations of AARC levels (see Table 3, values above the diagonal), all intercorrelations were positive. Higher perceived gains are thus related with higher perceived losses. For general AARC gains and losses, this correlation was *r* = 0.31. General AARC gains were strongly related with all domain-specific gains, and similarly, general AARC losses revealed strong associations with domain-specific losses (all *r* > 0.67). Generally, and not surprisingly, levels within gains and within losses were more strongly interrelated (all *r* > 0.35) than were gain levels with loss levels across the different domains (0.07 ≤ *r* ≤ 0.38).

**Table 3:** Associations between AARC domains.

	**1**	**2**	**3**	**4**	**5**	**6**	**7**	**8**	**9**	**10**	**11**	**12**
1 AARC Gains		0.77***	0.83***	0.82***	0.87***	0.82***	0.31***	0.19***	0.25***	0.23***	0.29***	0.26***
2 AARC Gains Health and Functioning	0.56***		0.56***	0.50***	0.56***	0.54***	0.25***	0.15**	0.23***	0.17**	0.23***	0.20***
3 AARC Gains Cognitive Functioning	0.57***	0.45***		0.64***	0.72***	0.55***	0.38***	0.25***	0.23***	0.28***	0.38***	0.36***
4 AARC Gains Interpersonal Relations	0.48***	0.41***	0.48***		0.69***	0.57***	0.30***	0.17***	0.25***	0.21***	0.29***	0.26***
5 AARC Gains Social-Cognitive/Social-Emotional Functioning	0.12*	0.30***	0.36***	0.45***		0.69***	0.21***	0.13**	0.15**	0.16**	0.20***	0.16**
6 AARC Gains Lifestyle and Engagement	0.62***	0.42***	0.42***	0.48***	0.43***		0.15**	0.07	0.16**	0.16**	0.11*	0.09
7 AARC Losses	0.18***	0.21***	0.31***	0.29***	0.22***	0.08		0.79***	0.77***	0.67***	0.80***	0.87***
8 AARC Losses Health and Functioning	0.15**	0.16**	0.26***	0.23***	0.18***	0.06	0.80***		0.50***	0.35***	0.49***	0.67***
9 AARC Losses Cognitive Functioning	−0.08	0.07	0.06	0.06	0.09	0.03	0.41***	0.17**		0.41***	0.53***	0.50***
10 AARC Losses Interpersonal Relations	0.12*	0.10*	0.23***	0.22***	0.13*	0.10	0.60***	0.32***	0.24***		0.48***	0.55***
11 AARC Losses Social-Cognitive/Social-Emotional Functioning	0.12*	0.18***	0.22***	0.25***	0.21***	0.05	0.78***	0.47***	0.34***	0.37***		0.67***
12 AARC Losses Lifestyle and Engagement	−0.02	0.08	0.14**	0.16**	0.15**	0.02	0.66***	0.44***	0.26***	0.39***	0.46***	

*Values above the diagonal correspond to the correlations between intercepts, values below the diagonal correspond to correlations between slopes.*

*p <0.05; **p <0.01; ***p <0.001.

Associations between AARC changes (see Table 3, values below the diagonal) were generally weaker than those between AARC levels, but associations were again—apart from two exceptions pointing at very small negative associations—consistently positive. Steeper declines in AARC gains over time thus seem to be associated with steeper declines in AARC losses. However, several correlations were small and below 0.20. For instance, the association between change in general AARC gains and change in general AARC losses was *r* =0.18. Change in general AARC gains was positively related with change in domain specific gains (0.12 ≤ r ≤ 0.62), and the same was true—with stronger intercorrelations—for change in general and domain-specific losses (0.41 ≤ r ≤ 0.80). The strongest change correlations were found between general AARC losses and AARC losses in health (*r* = 0.80) as well as between general AARC losses and AARC losses in social-cognitive/social-emotional functioning (*r* = 0.78). Changes within gains (0.12 ≤ *r* ≤ 0.62), and within losses (0.17 ≤ *r* ≤ 0.80) were more closely interrelated than changes in gain-loss pairings (−0.02 ≤ *r* ≤ 0.31). The changes in gains with regard to lifestyle and engagement were not significantly related with changes in any loss domain, and the changes in losses related to cognitive functioning were not significantly related with changes in any gain domain. Generally, our findings do not support the assumption of a dedifferentiated trend of very strongly coupled AARC changes.

### The Role of Personality and Age Stereotypes for Trajectories of General AARC Gains and Losses

**Figure 3:**
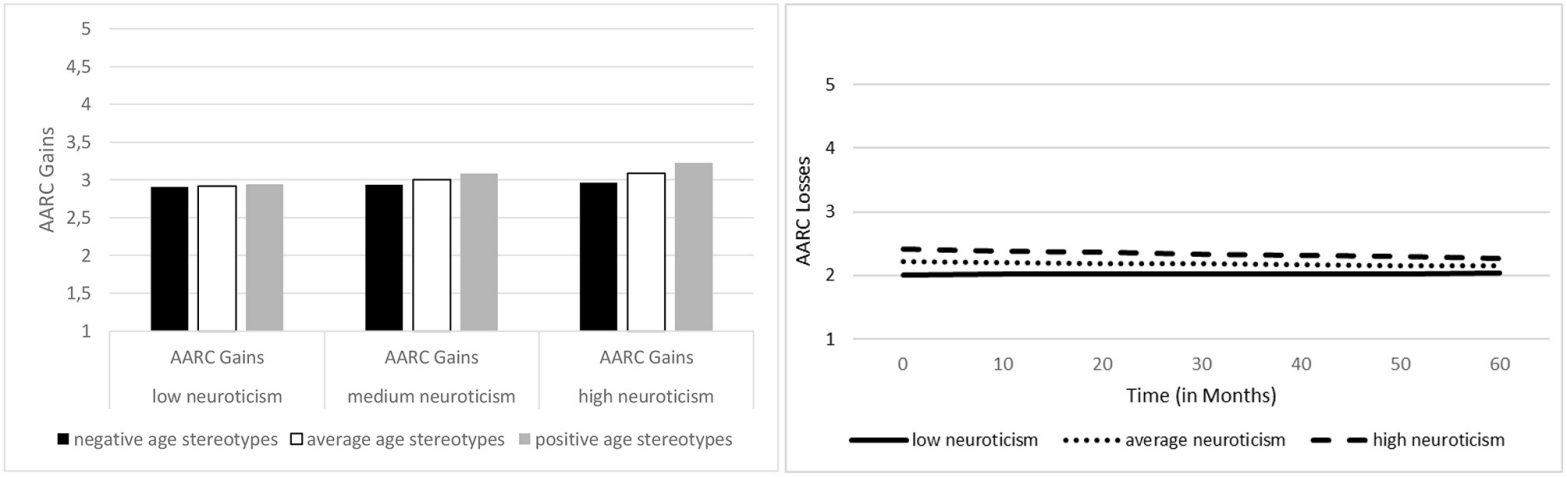
Associations of neuroticism with general AARC gains, by age stereotypes, and with general AARC losses. Left Figure: Both greater neuroticism and more positive age stereotypes predicted higher general AARC gain scores at baseline. Additionally, the interaction of neuroticism with age stereotypes reached statistical significance, indicating that the association between greater neuroticism and higher AARC gain scores was stronger among individuals who had more positive age stereotypes. Right Figure: Individuals scoring higher on neuroticism reported greater general AARC losses at baseline, but, at the same time, decline in AARC losses over time was also steeper among those scoring higher on neuroticism.

When investigating the role of personality traits and age stereotypes as well as their interactions for the prediction of AARC gains and losses, we retained—for the sake of model parsimony—only those personality-stereotype interaction terms in the models that reached statistical significance. Regarding the associations of personality and age stereotypes with general AARC gains, higher neuroticism, higher openness, higher conscientiousness, and more positive age stereotypes were significantly related with more perceived AARC gains at baseline (see Table 4). Moreover, there was a significant interaction of neuroticism with age stereotypes: The relationship of higher Neuroticism with more AARC gains at baseline was stronger when age stereotypes of an individual were more positive (see Figure 3). Neither personality traits nor age stereotypes (nor their interactions) were predictors of changes in general AARC gains over time.

**Table 4:** Longitudinal multilevel regression models: personality and age stereotypes as predictors of changes in AARC gains and losses.

**Model Estimates**	**AARC Gains (General)**	**AARC Losses (General)**
**Fixed Regression Coefficients**		
Intercept [*SE*]	3.002*** [0.056]	2.210*** [0.041]
Age [SE]	0.008* [0.003]	0.013*** [0.001]
Gender [SE]	0.116 [0.070]	−0.030 [0.052]
Education [SE]	0.023 [0.017]	0.007 [0.013]
Self-Rated Health [SE]	0.033 [0.042]	0.218*** [0.031]
Age Stereotypes [SE]	0.077* [0.036]	−0.086** [0.027]
Neuroticism [SE]	0.141* [0.059]	0.332*** [0.044]
Extraversion [SE]	0.107 [0.066]	−0.041 [0.049]
Openness [SE]	0.147* [0.074]	−0.071 [0.055]
Agreeableness [SE]	0.017 [0.077]	0.085 [0.057]
Conscientiousness [SE]	0.186** [0.067]	−0.059 [0.050]
Age Stereotypes × Neuroticism [SE]	0.103* [0.049]	
Linear slope 2012–2020 [*SE*]	−0.004*** [0.001]	−0.001 [0.001]
Age × Slope [SE]	−0.000* [0.000]	−0.000* [0.000]
Gender × Slope [SE]	0.001 [0.001]	−0.001 [0.001]
Education × Slope [SE]	−0.001* [0.000]	−0.000 [0.000]
Health × Slope [SE]	0.002* [0.001]	0.000 [0.001]
Age Stereotypes × Slope [SE]	−0.000 [0.001]	−0.001 [0.001]
Neuroticism × Slope [SE]	0.000 [0.002]	−0.002* [0.001]
Extraversion × Slope [SE]	−0.000 [0.001]	0.000 [0.001]
Openness × Slope [SE]	−0.001 [0.001]	0.002 [0.001]
Agreeableness × Slope [SE]	0.001 [0.001]	0.001 [0.001]
Conscientiousness × Slope [SE]	−0.002 [0.001]	−0.001 [0.001]
**Random Variances**		
Variance Intercept [*SE*]	0.217*** [0.027]	0.113*** [0.015]
Variance Linear Slope [*SE*]	0.000 [0.000]	0.000 [0.000]
Cov. Intercept-Slope [*SE*]	0.001 [0.000]	0.000 [0.000]
Residual Variance [*SE*]	0.151*** [0.012]	0.094*** [0.008]
BIC	1,781.3	1,265.2
*R* ^2^	0.119	0.109

*Time unit is months (since 2012). R^2^ was computed according to Xu (83). AARC, Awareness of age-related change.*

*p <0.05; **p <0.01; ***p <0.001.

General AARC loss scores at baseline were higher among individuals with more negative stereotypes, as well as among those scoring higher on neuroticism (see Table 4 and Figure 3). No other personality trait was significantly related with general AARC losses, and no significant personality-age stereotypes interactions emerged. With regard to change in AARC losses, higher neuroticism—though associated with higher AARC losses at baseline—predicted a steeper decrease in AARC losses over time (see Figure 3).

## Discussion

Research on views on aging needs to better consider the multidimensionality of views of aging and the multidirectionality of their changes over time (33). In this study, we therefore investigated trajectories of awareness of age-related change (AARC) over up to ~5 years, including overall gains and losses as well as gains and losses across 5 subdomains (health and physical functioning, cognitive functioning, interpersonal relations, social-cognitive and socio-emotional functioning, lifestyle and engagement). We also analyzed how personality traits and age stereotypes, as well as their interactions, are associated with AARC and changes thereof.

### Developmental Trajectories of AARC Gains and Losses

Controlling for age, gender, education, and self-rated health, we found that overall AARC gains, as well as all AARC gain subdomains—except gains in interpersonal relations, revealed a mean-level decline. For AARC losses, a significant mean-level decline was observed only for losses in interpersonal relations and in lifestyle and engagement, whereas all other domains remained, on average, stable over time. Decreasing AARC gains over time have been reported before (38), also in very old adults (35), whereas stability in AARC losses is not in line with the available prior evidence. However, as pointed out before, prior longitudinal findings on AARC trajectories are scarce and limited to overall AARC gains and losses, rather than to AARC subdomains.

Across most domains, individuals thus tend to perceive decreasing gains over time, although this trend is—at least from a mean-level perspective—not accompanied by perceiving increasing losses over time. It might be the case that decreasing gains are particularly salient in later life, whereas losses occur more gradually over time and are maybe anticipated to a stronger extent by aging individuals than decreasing gains. The relation of changes and their attribution to age might also be rather complex and change over time (50). The only domain not revealing a mean-level gain decline, but at the same time revealing a mean-level loss decline, is the one of interpersonal relations. This domain could thus be a particularly “loss-resilient” resource (53, 54).

It could be argued that such mean-level trends are misleading, as they do not take differences in change according to baseline age into account. Indeed, we found that among those who were older at baseline, decreases in general AARC gains and in gains related to cognitive functioning and lifestyle and engagement were steeper; however, also AARC losses in cognitive functioning revealed a steeper decrease over time with advancing chronological age. Thus, at least across some domains, decreases in perceptions of gains are more pronounced with increasing age, which might reflect objective age-related declines in domains such as cognitive functioning [e.g., (41, 46, 85)]. However, the pattern of AARC changes and their association with chronological age is even more complex. For instance, not only perceived gains in cognitive functioning decreased to a stronger extent among individuals who are older, but also perceived losses in the same domain. Moreover, whereas most AARC gain domains revealed a trend toward decline over time, baseline age differences in overall AARC gains as well as in AARC gains regarding health and functioning were in favor of older adults. Baseline age and gains in lifestyle and engagement were non-linearly associated, with highest scores among young-old individuals and lower scores among middle-aged and (very) old adults. This finding might reflect the so-called “honeymoon effect” (86, 87) among individuals who just retired from work and now perceive plenty of options with regard to lifestyle and leisure activities. Baseline age and AARC loss domains were positively interrelated (except for the age association with losses in social-cognitive/socio-emotional functioning, which was not significant), that is, with advancing age individuals perceived more losses across the different domains. This age effect was particularly striking with regard to losses in interpersonal relations which were non-linearly associated with age and thus seem to accumulate in old and very old age. This is in line with findings of an increased prevalence of loneliness in very old age [e.g., (88, 89)], although reported findings are not entirely consistent, and not all studies confirm such an age trend in loneliness [e.g., (90)]. This age pattern also calls into question our interpretation of the “age resilience” with regard to interpersonal relations: Although perceived gains in interpersonal relations remained, on average, stable over time, and perceived losses in this domain even decreased, levels of AARC losses in interpersonal relations were higher among individuals who were older.

This complex pattern of divergent trends of longitudinal change in AARC vs. cross-sectional age differences in AARC at baseline underlines the importance and necessity to investigate AARC longitudinally: The age group differences in several AARC gains that are in favor of older adults should not be misinterpreted as evidence for longitudinal increase in AARC gains over time, as we actually observed mean-level decreases in most AARC gain domains over time. Similarly, the positive associations between age and most AARC loss domains at baseline are in contrast to the observed stable or decreasing AARC losses over time. Age differences in AARC gains and losses could thus reflect cohort rather than aging effects. According to Beyer et al. (91), there has indeed been a secular change toward less loss-oriented views on aging, which is in line with the positive cross-sectional association of age with AARC losses—if interpreted as a cohort effect—that we observed. However, Beyer et al. (91) also report a cohort effect toward more gain-oriented views on aging in terms of perceptions of continuous growth, which is in contradiction to our observation of higher baseline AARC gains across several domains among individuals who were older at baseline and thus represent earlier-born birth cohorts. Also, Wahl et al. (92) did not find any cohort differences in attitude toward own aging. Available findings with regard to cohort trends in views on aging are thus scarce and not consistent, and more research is required.

Another explanation for the discrepancy of longitudinal change trends vs. cross-sectional age differences in our study could be that 5 years might not be sufficient to observe a meaningful increase in AARC losses. The overall stability or even decrease AARC loss domains could also imply that middle-aged and older adults might be successful in preventing (perceived) losses. The underlying reason or mechanism might be that from midlife to older age, the general goal focus shifts, due to changes in the overall gains-losses ratio, toward prevention of loss, rather than toward increases of gains (18, 93), which might also explain why decreases in AARC gains over time were observed in this study.

### Intercorrelations of AARC Levels and Changes

However, mean-level changes in AARC gains and losses are by no means sufficient for a comprehensive understanding of dynamics in AARC, and particularly of couplings across AARC changes. Therefore, we additionally investigated to what extent baseline scores across all AARC gain and loss domains, as well as their changes over time are interrelated. Little evidence was found for an “AARC dedifferentiation” in terms of uniform and strongly interrelated changes across the different AARC domains: Baseline AARC gains and baseline AARC losses revealed stronger associations than changes in AARC gains and AARC losses. Not surprisingly, levels and changes within AARC gain domains and within loss domains were more strongly interrelated than correlated changes of gain-loss constellations. Finally, both levels and changes of gains and losses were mostly positively, though overall weakly interrelated. That is, individuals scoring higher on certain AARC gain domains tended to score also higher in AARC loss domains, which is in line with prior research (21, 22). In addition, individuals revealing steeper decreases in AARC gain domains also tended to reveal steeper decreases in AARC loss domains. There might thus be an overall sensitivity to age-related change, and those who reveal such a higher sensitivity score higher on both AARC losses and gains, whereas individuals who are less sensitive and attentive toward age-related changes score lower on both AARC losses and gains. However, none of the correlations between intercepts or slopes of gain-loss pairings, all adjusted for age, gender, education, and self-rated health, reached a large effect size [i.e., all *r* < 0.50 (94)], and only one correlation of change in AARC gains with change in AARC losses reached a medium effect size (*r* ≥ 0.30). AARC gains and losses are thus to a large extent independent of each other, and their changes do not follow a pattern of dedifferentiation and strong coupling. A stronger coupling seems to exist between overall AARC loss changes and changes in the different AARC loss domains than between overall AARC gain changes and changes in the AARC gain domains. Perceiving change in general AARC losses over time might thus “spread” across the different AARC domains which are all perceived to change, whereas such a coupling is less pronounced within AARC gains.

### The Role of Personality and Age Stereotypes

With regard to associations between personality traits and general AARC gains and losses, we found that higher neuroticism scores were associated with both greater AARC gains and greater AARC losses at baseline. Additionally, higher openness and conscientiousness were associated with greater AARC gains at baseline. Only one personality trait reached statistical significance as predictor of AARC changes, namely neuroticism, with greater neuroticism predicting a steeper decline in AARC losses. These findings largely correspond to the findings reported by Rupprecht et al. (60), which were based on the same data set, as well as to findings relating personality to other views on aging domains (72, 73). However, we were not able to replicate the association of conscientiousness with change in AARC losses reported by Rupprecht et al. (60); instead we found a longitudinal association of greater neuroticism with steeper decline of AARC losses. This discrepancy could be due to different statistical approaches, to a different set of covariates and predictors (for instance, we additionally included age stereotypes as predictors) as well as to a different number of measurement occasions [we included one additional measurement occasion compared to Rupprecht et al. (60)].

Generally, our findings suggest that associations of personality with AARC mostly emerge on a cross-sectional level, whereas only neuroticism was significantly related with change in AARC losses. The role of neuroticism is particularly interesting and complex, as greater neuroticism was related with both greater AARC gains and AARC losses at baseline. Additionally, it was related with a greater decrease in AARC losses over time. Rupprecht et al. (60) discuss in this context that “This association [between neuroticism and AARC gains] may stem from tendencies toward caution and carefulness in individuals scoring high in neuroticism which may resonate with aspects of AARC gains (e.g., ‘With my increasing age, I realize that I pay more attention to my health')” (p. 64). There might thus be a form of “healthy neuroticism” (95, 96), such as high neuroticism coupled with high conscientiousness, that promotes AARC gains and declines in AARC losses over time. This might be an interesting avenue for further research. Given that greater neuroticism is associated with greater attention to one's own health—which might not only result in greater symptom reporting (67), but also in more frequent medical consultations, check-ups and greater general caution with regard to potential health risks-, such behaviors associated with neuroticism might contribute to reduce AARC losses over time. Alternatively, individuals who score high on neuroticism might also be particularly pessimistic with regard to anticipated age-related changes. When having reached a certain age, these individuals might be surprised that not all anticipated age-related losses have set in, so that their individually perceived AARC losses decrease over time.

Finally, the role of neuroticism for AARC gains was found to be moderated by age stereotypes. With increasing positivity of these stereotypes, the positive associations between neuroticism and AARC gains at baseline was stronger. Neuroticism might thus have a stronger positive impact on AARC gains when an individuals' general age stereotypes are positive, whereas negative age stereotypes mitigate this impact. However, given that this moderating role of age stereotypes was only found for the cross-sectional association between AARC gains and neuroticism, conclusions regarding cause and effect cannot be drawn. It is also important to point out that this interaction effect of personality with age stereotypes was the only one out of 10 potential interactions that reached statistical significance. Associations of personality and age stereotypes with AARC thus seem to be to a large extent independent of each other.

Regarding the role of *age stereotypes*, we found that more negative age stereotypes were associated with greater AARC losses and fewer AARC gains at baseline, which corresponds to the predictions of stereotype embodiment theory (74) and to other empirical findings (22, 50), although there were no significant longitudinal effects of age stereotypes on AARC changes. Our measurement interval might have been too short to detect such effects. Further research is thus needed to investigate which age stereotypes affect within-person trajectories in views on aging under which circumstances [cf. (50)].

### Strengths and Limitations

Research on long-term changes in *multidimensional* views on aging is still scarce. With this study, we aimed to address this research gap by investigating AARC changes in middle-aged and older individuals over a period of about 5 years, with an explicit distinction between all AARC subdomains. Moreover, this is, to our knowledge, the first study analyzing a multidimensional concept of views on aging in parallel with both personality and age stereotypes, as well as their interaction.

Apart from these strengths, our study is also subject to several limitations. We restricted our measurement interval to about 5 years, comprising 3 measurement occasions. Even though we had a fourth measurement from 2020 at our disposal, we decided not to include it, as it might have been affected by a COVID-19 period effect (38, 79). Moreover, only a short-form of the AARC scale [AARC-10 SF (25)] was included in the 2020 measurement occasion, so that AARC subdomains were not covered. In addition, there was a shift in assessment mode in 2020, from paper-pencil questionnaires to an online survey (at least for the majority of study participants), which might bias estimate of AARC changes between 2012 and 2020. Having only three measurement occasions at our disposal, we were not able to model potential non-linear changes in AARC. Also, our observation period might have been too short to detect an accumulation of AARC losses over time and to identify further effects of personality or age stereotypes on AARC changes. Therefore, future studies on AARC changes based on longer time intervals and more measurement occasions are desirable.

Further, although the original construction of the different AARC subscales were conceptually and empirically driven (14, 16), note that a 10-factor solution has not been confirmed in prior studies. Hence, caution is in place with respect to the interpretation of our domain-specific findings. However, if there was a very high overlap between the AARC domains, and if the AARC domains did not differentiate between different aspects of perceived age-related change, stronger associations between the levels, and particularly the changes, of the AARC domains should have resulted. However, we did not find evidence of strong couplings among the different AARC subdimensions and of an “AARC dedifferentiation.”

The very limited effects of personality and age stereotypes on AARC changes might to some extent be due to our very broad approach of using the Big Five personality traits and general age stereotypes. Personality facets, rather than overall traits, as well specific stereotype domains, rather than general age stereotypes, might be stronger predictors of AARC changes. Also, testing the stereotype-matching hypothesis (97) by linking specific stereotype domains (e.g., regarding health) with congruent AARC subdomains, rather than general age stereotypes and overall AARC gains and losses, might result in stronger longitudinal effects of age stereotypes on AARC, which requires further investigation. Besides, internal consistency (Cronbach's α) of some personality scales was low, so that the effects of personality need to be interpreted with caution. However, Cronbach's α provides a conservative estimation of reliability, and low α scores do not necessarily indicate a lack of reliability, but might also reflect (particularly in the case of openness) that a scale or construct is not unidimensional. Finally, personality traits and age stereotypes are to some extent plastic and changeable (38, 75), also in old and very old age (44, 98, 99), so that changes in personality, age stereotypes and views on aging might be interrelated [see for instance (72)], maybe more strongly than are associations of personality and age stereotypes when considered as time-invariant and thus measured at only one point in time with change in AARC or in other views on aging domains.

## Conclusion

Our study shows that how individuals perceive their aging process and the changes that are attributed to this process is highly dynamic, depends on the valence (gain vs. loss) and the specific domain of change, as well as on individuals' age, personality and age stereotypes. Overall, our findings regarding the differences in AARC gain and loss trajectories support the conceptualization of views on aging as a multidimensional and multidirectional construct: Whereas a decline in perceived age-related gains—at least in some domains—might be unavoidable for many middle-aged and older adults, individuals seem to be good at “loss management” and to some extent resilient against increases in AARC losses. This knowledge might be used when developing interventions to change views on aging in later life. Future studies also need to take these complexities into account when investigating and disentangling age differences vs. changes of views on aging across the life span.

## Data Availability Statement

The dataset presented in this article is not readily available; explicit informed consent from all study participants is, according to the general data protection regulation of the European Union (100), according to German law (101), and according to the ethical principles of the Declaration of Helsinki, mandatory when ever data—even if deidentified—are made available to persons beyond the project team. We also received a clear statement from the Institutional Ethics Review Board of the Faculty of Behavioral and Empirical Cultural Sciences of Heidelberg University, according to which we need explicit consent from study participants to make any data available. Moreover, it is important to point out that absolute deidentification may no longer be warranted as soon as information on only a few sociodemographic variables is available [e.g., (102)]. The “Rat für Wirschafts- und Sozialdaten” [German Data Forum; (103)] states that whenever it is planned and necessary to forward data to research partners, repositories, and other institutions with the purpose of joint use, archiving, or publishing of anonymized data, this needs to be stated already within the informed consent for study participant (p. 27; original citation in Germany language: “Auch eine möglicherweise geplante und notwendige Weitergabe von Daten an Forschungspartner, Repositorien und andere Stellen zum Zwecke der gemeinsamen Bearbeitung, einer Archivierung bzw. Nachnutzung oder einer Veröffentlichung in anonymisierter Form ist bereits in der Einwilligung zu verankern”). The informed consent text which the study participants received in this study was quite restrictive and therefore leaves us no permission to share the data, as study participants were assured that their data will *under no circumstances* be forwarded to any persons who are not involved in this study project. This was a formulation that was recommended in order to give study participants the highest possible control over their data. Sharing the data with researchers via repositories or any other formats would thus be in a clear contradiction to what we communicated to the study participants. Two years after having carried out the study, it is simply not possible to recontact all study participants so as to ask for their consent to share and store the data in a repository. Therefore, there is, unfortunately, no option for data sharing available to us that would not violate German or European data protection law. Still, we declare that we are ready to fully collaborate with scholars aiming to replicate our findings. For example, we are ready to rerun in full transparency in a respective meeting with an interested scholar, online or face-to-face, our original data analytical code with the data on which this article is based, to show the findings, as well as to run additional analyses as requested by such a scholar.

## Ethics Statement

The studies involving human participants were reviewed and approved by Institutional Ethics Review Board of the Faculty of Behavioral and Empirical Cultural Sciences of Heidelberg University. The patients/participants provided their written informed consent to participate in this study. Approval for Wave 4 of the longitudinal data used in this study was received from the Institutional Ethics Review Board of the Faculty of Behavioral and Empirical Cultural Sciences of Heidelberg University (AZ Wahl 2020 1/1). Wave 3 was approved by the same board with a letter dating from February 17, 2017 (no protocol no.). Waves 1 and 2 were approved by the Colorado State University (CSU) Institutional Review Board (IRB) protocol #10-2080H based on a formal cooperation between Heidelberg University and CSU.

## Author Contributions

MW computed all statistical analyses and wrote the results section. AK, MW, and H-WW conceptualized the study (introduction section), wrote the discussion section, and contributed to all other parts of the manuscript. All authors were involved in writing the article and approved the submitted version.

## Funding

The data collection for this study was made possible by funding from the Carl Zeiss Foundation (Project P2017-01-002; HEIAGE, Heidelberg University, Germany) and a Humboldt Research Award to Manfred Diehl and Hans-Werner Wahl based on their Transcoop funding scheme.

## Conflict of Interest

The authors declare that the research was conducted in the absence of any commercial or financial relationships that could be construed as a potential conflict of interest.

## Publisher's Note

All claims expressed in this article are solely those of the authors and do not necessarily represent those of their affiliated organizations, or those of the publisher, the editors and the reviewers. Any product that may be evaluated in this article, or claim that may be made by its manufacturer, is not guaranteed or endorsed by the publisher.
