# Supplementary material for: Awareness of Age-Related Changes Among Middle-Aged and Older Adults: Longitudinal Trajectories, and the Role of Age Stereotypes and Personality Traits
Source: Front Psychiatry. 2022 May 25;13:902909. doi: 10.3389/fpsyt.2022.902909 (PMC9174521; doi:10.3389/fpsyt.2022.902909)
Supplement: Supplementary file 1 [file Data_Sheet_1.docx]

Awareness of Age-Related Changes Among Middle-Aged and Older Adults: Longitudinal Trajectories, and the Role of Age Stereotypes and Personality Traits

Markus Wettstein, Anna E, Kornadt, Hans-Werner Wahl

**Online Supplement**

**Supplemental Table 1**

*Sample Description (Baseline)*

|  | *M*±*SD* or *n* (%) | | |  |  |
| --- | --- | --- | --- | --- | --- |
| Age (2020) | 62.94±11.84 | | |  |  |
| Female | 272 (64.3%) | | |  |  |
| Education (years) | 11.53±1.96 | | |  |  |
| Subjective Health ^a^ | 2.76±0.82 | | |  |  |
| Age Stereotypes ^b^ | 4.91±0.90 | | |  |  |
| Neuroticism | 2.53±0.62 | | |  |  |
| Extraversion | 3.28±0.53 | | |  |  |
| Openness | 3.49±0.44 | | |  |  |
| Agreeableness | 3.71±0.43 | | |  |  |
| Conscientiousness | 3.86±0.47 | | |  |  |
|  | T1 (2012) | | | T2 (2015) | T3 (2017) |
|  | | *M*±*SD* or *n* (%) | | *M*±*SD* or *n* (%) | *M*±*SD* or *n* (%) |
| AARC Gains (Cronbach’s α T1-T3: .92, .92, .93) | | | 3.09±0.65 | 2.89±0.70 | 2.89±0.70 |
| AARC Losses (Cronbachs’s α T1-T3: .92, .92, .93) | | | 2.23±0.61 | 2.09±0.59 | 2.15±0.61 |
| AARC Gains Health and Functioning (Cronbach’s α T1-T3: 80, .76, .81) | | | 3.03±0.88 | 2.74±0.83 | 2.76±0.88 |
| AARC Gains Cognitive Functioning (Cronbach’s α T1-T3: .75, .78) | | | 3.05±0.72 | 2.73±0.77 | 2.76±0.78 |
| AARC Gains Interpersonal Relations (Cronbach’s α T1-T3:.71, .73, .75) | | | 2.79±0.77 | 2.77±0.82 | 2.74±0.81 |
| AARC Gains Social-Cognitive/Social-Emotional Functioning (Cronbach’s α T1-T3:.80, .81, .80) | | | 3.35±0.85 | 3.18±0.86 | 3.19±0.84 |
| AARC Gains Lifestyle and Engagement (Cronbach’s α T1-T3: .84, .86, .84) | | | 3.26±0.93 | 3.01±1.02 | 3.01±0.97 |
| AARC Losses Health and Functioning (Cronbach’s α T1-T3: .86, .87, .87) | | | 2.69±0.91 | 2.61±0.90 | 2.73±0.89 |
| AARC Losses Cognitive Functioning (Cronbach’s α T1-T3: .86, .87, .87) | | | 2.32±0.85 | 2.14±0.77 | 2.17±0.76 |
| AARC Losses Interpersonal Relations (Cronbach’s α T1-T3: .73; .75; 78) | | | 1.55±0.59 | 1.43±0.54 | 1.47±0.60 |
| AARC Losses Social-Cognitive/Social-Emotional Functioning (Cronbach’s α T1-T3: .72, .74, .73) | | | 2.14±0.72 | 2.04±0.73 | 2.07±0.72 |
| AARC Losses Lifestyle and Engagement (Cronbach’s α T1-T3: 69, .67, .71) | | | 2.14±0.72 | 2.04±0.73 | 2.07±0.72 |

*Note. M* = mean; *SD* = standard deviation.

^a^ Lower values indicate better health.

^b^ Higher scores indicate more positive age stereotypes.

**Supplemental Figure 1: Associations of AARC Gains in Lifestyle and Engagement and of Losses in Interpersonal Relations with Age**


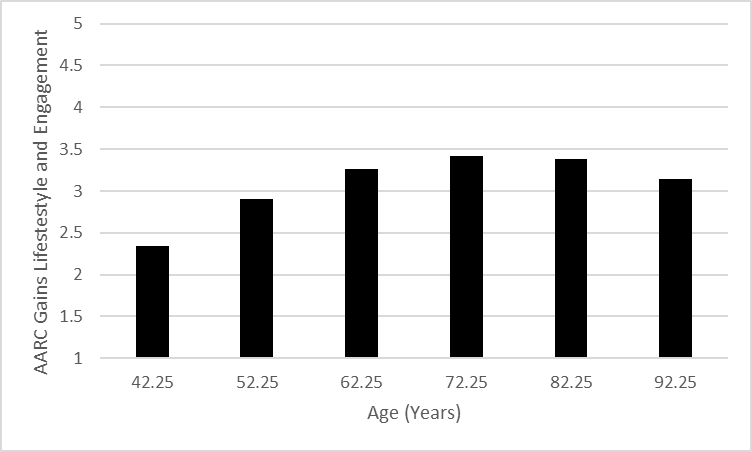

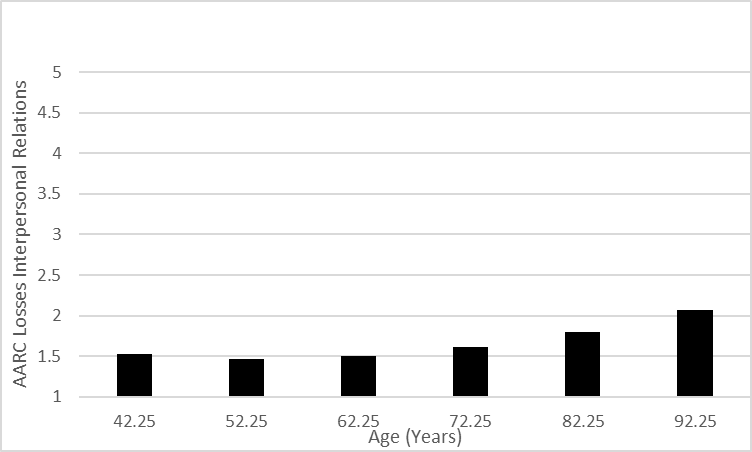


*Note.* The association of age with baseline scores on AARC gain scores in lifestyle and engagement is nonlinear, with gains reaching their peak in early old age, whereas they are lower in midlife and very old age. There is also a nonlinear age trend with regard to AARC losses in interpersonal relations: Baseline loss scores are particularly high among oldest-old individuals.
